# Efficacy and Safety of Telavancin in Clinical Trials: A Systematic Review and Meta-Analysis

**DOI:** 10.1371/journal.pone.0041870

**Published:** 2012-08-16

**Authors:** Konstantinos A. Polyzos, Michael N. Mavros, Konstantinos Z. Vardakas, Marinos C. Makris, Petros I. Rafailidis, Matthew E. Falagas

**Affiliations:** 1 Alfa Institute of Biomedical Sciences (AIBS), Athens, Greece; 2 Department of Medicine, Henry Dunant Hospital, Athens, Greece; 3 Department of Medicine, Tufts University School of Medicine, Boston, Massachusetts, United States of America; Los Angeles Biomedical Research Institute, United States of America

## Abstract

**Introduction:**

The epidemiology and antibiotic resistance of *Staphylococcus aureus* have evolved, underscoring the need for novel antibiotics, particularly against methicillin-resistant *S. aureus* (MRSA). Telavancin is a bactericidal lipoglycopeptide with potent activity against Gram-positive pathogens.

**Objective:**

To systematically review and synthesize the available evidence from randomized controlled trials (RCTs) evaluating telavancin in the treatment of patients with infections due to Gram-positive organisms with the methodology of meta-analysis.

**Results:**

Six RCTs comparing telavancin with vancomycin were included; 4 (2229 patients) referred to complicated skin and soft tissue infections (cSSTIs) and 2 (1503 patients) to hospital-acquired pneumonia (HAP). Regarding cSSTIs, telavancin and vancomycin showed comparable efficacy in clinically evaluable patients (odds ratio [OR] = 1.10 [95% confidence intervals: 0.82–1.48]). Among patients with MRSA infection, telavancin showed higher eradication rates (OR = 1.71 [1.08–2.70]) and a trend towards better clinical response (OR = 1.55 [0.93–2.58]). Regarding HAP, telavancin was non-inferior to vancomycin in terms of clinical response in two Phase III RCTs; mortality rates for the pooled trials were comparable with telavancin (20%) and vancomycin (18.6%). Pooled data from cSSTIs and HAP studies on telavancin 10 mg/kg indicated higher rates of serum creatinine increases (OR = 2.22 [1.38–3.57]), serious adverse events (OR = 1.53 [1.05–2.24]), and adverse event-related withdrawals (OR = 1.49 [1.14–1.95]) among telavancin recipients.

**Conclusion:**

Telavancin might be an alternative to vancomycin in cases of difficult-to-treat MRSA infections. The potent antistaphylococcal activity of telavancin should be weighted against the potential for nephrotoxicity.

## Introduction

Antimicrobial resistance of *Staphylococcus aureus* poses a major threat to public health [1]. In 2005, methicillin-resistant *S. aureus* (MRSA) in the United States (US) accounted for 48% and 60% of *S. aureus* isolates from outpatients and inpatients, respectively [2]. Strains of vancomycin-intermediate *S. aureus* (VISA), heteroresistant VISA (hVISA) and vancomycin-resistant *S. aureus* (VRSA) have also emerged leading to an increase in vancomycin treatment failures [3]–[8], while newer antibiotics have not been superior to vancomycin in double-blind randomized controlled trials (RCTs) [9]. This shift in *S. aureus* susceptibility, along with an increase in community-acquired MRSA infections [10], underscore the need for novel antimicrobial agents with potent antistaphylococcal activity [11].

Telavancin, a semisynthetic derivative of vancomycin (VAN), is a member of the lipoglycopeptide class of antibiotics. It is a bactericidal concentration-dependent antibiotic with a dual mechanism of action, involving inhibition of cell wall synthesis and disruption of membrane integrity [12]. Telavancin exhibits potent in vitro activity against a variety of Gram-positive organisms, including *S. aureus* (MRSA, hVISA, VISA), coagulase-negative staphylococci and *Streptococcus spp*. It is also active against VAN-susceptible and VanB VAN-resistant enterococci, as well as various Gram-positive anaerobic organisms [13]–[15]. In the US and Canada, telavancin is approved for the treatment of patients with complicated skin and soft tissue infections (cSSTIs). In Europe, this drug has been approved for hospital-acquired pneumonia (HAP), including ventilator-associated pneumonia (VAP), caused by MRSA, when other alternatives are not suitable [16].

In this study, we systematically reviewed the available clinical and microbiologic outcomes of RCTs that compared the efficacy and safety of telavancin with that of other antibiotics, and pooled them, if possible, in a meta-analytic framework.

## Methods

### Data sources

PubMed, Scopus, Cochrane Central Register of Controlled Trials (Central) and LILACS databases were searched for relevant studies published up to March 2012. The search term applied was “telavancin” or “TD-6424”. Abstracts from the Interscience Conference on Antimicrobial Agents and Chemotherapy (ICAAC) (2005–2011) and the European Congress of Clinical Microbiology and Infectious Diseases (ECCMID) (2003–2012), as well as references of relevant articles, were hand-searched. No restrictions in the language or year of publication were imposed.

### Study selection

Three investigators (KAP, MNM and MCM) independently searched the literature and examined relevant studies for potential inclusion in this meta-analysis. To be considered eligible, a study should be an RCT examining the efficacy or safety of telavancin compared to any other antibiotic regimen for the treatment of patients with any type of infection. Animal studies and pharmacokinetic or pharmacodynamic studies were excluded. Additional antimicrobial agents (mainly those with effectiveness against Gram-negative pathogens involved in polymicrobial infections) could be used in the RCTs.

### Data extraction

Three reviewers (KAP, MNM and MCM) independently extracted the following data from each study: year of publication; study design; study population; number of patients (intention to treat [ITT], clinically evaluable [CE], microbiologically evaluable [ME], ME with *S. aureus* infection [ME-S. aureus], and ME with MRSA infection [ME-MRSA]); antimicrobial agents and doses used; clinical and microbiologic outcomes; and data on safety. Any disagreement was resolved by consensus in meetings that involved all authors. When trial data was not fully available, we contacted the corresponding authors asking for additional information or used conference reports and data reported to the US Food and Drug Administration and European Medicines Agency.

The ITT population comprised patients who received at least one dose of the study medication. The CE patients were those who met all inclusion and exclusion criteria and had a clinical response of either cure or failure; patients were excluded from this population if they had only a Gram-negative pathogen or a polymicrobial infection including a Gram-negative isolate resistant to aztreonam. The ME population was a subset of patients in the CE population with a Gram-positive isolate at baseline.

The quality of the included RCTs was evaluated with a modified Jadad score, which considers randomization, generation of random numbers, allocation concealment, details of double-blinding procedure and information on withdrawals [17]. One point was awarded for the specification of each criterion, with a maximum score of 5. A score higher than 2 points was used to denote a trial of good methodological quality.

### Analyzed outcomes

The primary outcome measures for this meta-analysis were treatment success (as defined in individual studies) in the ITT and CE populations, clinically significant increase in serum creatinine (defined as serum creatinine level ≥1.5 mg/dl at the end-of-therapy visit with an increase of at least 50% or 0.5 mg/dl over the pretreatment level), and all-cause mortality. Secondary outcomes comprised clinical success in ME-S. aureus and ME-MRSA patients, microbiologic eradication, time to clinical improvement or cure, overall adverse events (AE), serious AE [18], and AE-related withdrawals.

### Data analysis and statistical methods

Statistical analyses were performed using Review Manager [19]. Pooled odds ratios (OR) and 95% confidence intervals (95% CI) for all primary and secondary outcomes were calculated by use of the Mantel-Haenszel fixed effect (FEM) and random effects model (REM) as appropriate [20], [21]. Results from the FEM were presented only when there was no heterogeneity between studies; otherwise, results from the REM were presented. Heterogeneity was assessed by using both χ^2^ and I^2^ tests; p<0.1 (χ^2^) or I^2^≥40% were considered to denote significant heterogeneity. Publication bias was not assessed due to the small number of the included studies [22].

## Results

### Included studies and their main characteristics

**Figure 1:**
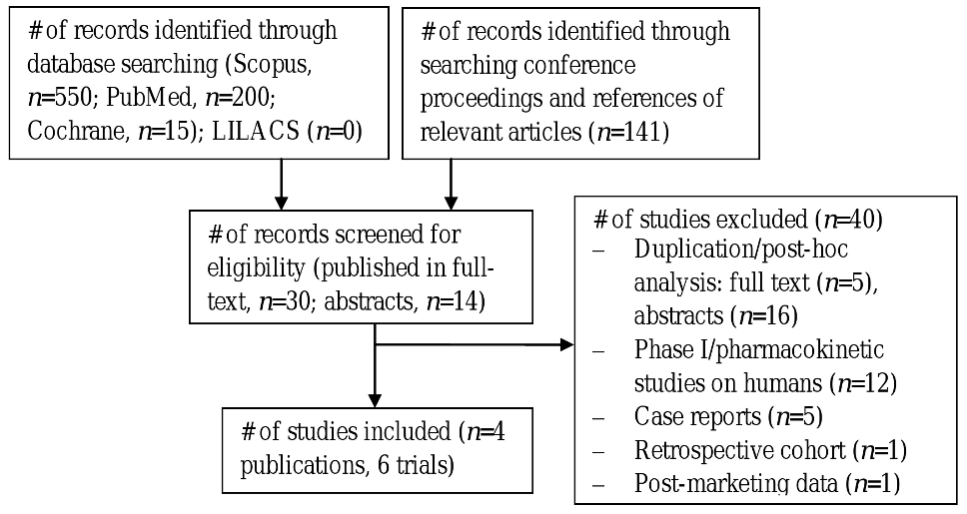
Flow diagram of the selection process of the included studies.

The study flow chart is presented in Figure 1. The database search generated a total of 765 records, whereas hand-searching of conference proceedings identified 141 additional records. After removing duplicates only 44 records were screened for eligibility, of which 40 were excluded. Overall, four publications describing 6 RCTs comparing telavancin with standard therapy were included in the systematic review [23]–[26]. Additional information was extracted from 2 abstracts [27], [28].

The main characteristics of the analyzed studies are shown in Table 1. All 6 RCTs were sponsored by the pharmaceutical industry and were multicenter double-blind trials (Jadad score ≥4) evaluating adult men or non-pregnant women with cSSTIs (2229 subjects) [23]–[25] or HAP (1503 subjects) [26] due to suspected or confirmed Gram-positive pathogens. Regarding cSSTIs, two Phase II (FAST) trials compared telavancin IV at 7.5 mg/kg/24 h or 10 mg/kg/24 h with standard therapy (vancomycin or antistaphylococcal penicillin, chosen prior to randomization) [23], [24], whereas two Phase III (ATLAS) non-inferiority trials compared telavancin IV at10 mg/kg/24 h with vancomycin [25]. Regarding HAP, two Phase III (ATTAIN) non-inferiority trials compared telavancin IV at 10 mg/kg/24 h with vancomycin [26]. The dose of vancomycin could be individually adjusted and could be switched to an antistaphylococcal β-lactam in case of infection due to methicillin-susceptible *S. aureus* [23]–[28].

**Table 1:** The main characteristics of the included studies.

Study [ref]	Study design (Jadad score)	Participants	Comparator^a^	Populations (pts)	Notes
FAST 1 [23]	MC DB Phase II RCT (4)	Men or non-pregnant women with cSSTIs (abscess, 48%; cellulitis, 37%; wound infection, 11%); mean age (yr): 44/44; US, South Africa, 2003–2004	VAN iv 1 g q12h: 76%; antistaph. PCN^b^ iv q6h: 24%	ITT: 84 vs 83; ITT-S. aureus: 50 vs 52; ITT-MRSA: 22 vs 26; CE: 72 vs 69; ME: 56 vs 56	CrCL<50 ml/min, QTcF>470 ms = exclusion; AZT: 35% vs 29%; MET: 24 vs 25%
FAST 2 [24]	MC DB Phase II RCT (4)	Men or non-pregnant women with cSSTIs (abscess, 58%; cellulitis, 29%; wound infection, 11%); mean age (yr): 45/42; US, South Africa, 2004	VAN iv 1 g q12h: 93%; antistaph. PCN^b^ iv q6h: 7%	ITT: 100 vs 95; CE: 77 vs 77; ME: 64 vs 57; ME-*S. aureus*: 50 vs 41; ME-MRSA: 26 vs 19	QTcF>500 ms = exclusion; AZT: 41% vs 37%; MET: 44% vs 38%
ATLAS 1 [25], [27]	MC DB Phase III RCT (5)	Men or non-pregnant women with cSSTIs (abscess, 44%; cellulitis, 37%; wound infection, 15%); mean age (yr): 49/48; multinational, 2005–2006	VAN iv 1 g q12h	ITT: 426 vs 429; CE: 346 vs 349; ME: 237 vs 255; ME-MRSA: 116 vs 138	Prespecified pooled analysis design; QTcF>500 ms = exclusion; AZT: 32% vs 33%; MET: 23% vs 22%
ATLAS 2 [25], [28]	MC DB Phase III RCT (5)	Men or non-pregnant women with cSSTIs (abscess, 41%; cellulitis, 37%; wound infection, 13%); mean age (yr): 49/50; multinational, 2005–2006	VAN iv 1 g q12h	ITT: 502 vs 510; CE: 399 vs 395; ME: 290 vs 281; ME-MRSA: 162 vs 163	
ATTAIN 1 [26]	MC DB Phase III RCT (5)	Men or non-pregnant women with HAP; mean age (yr): 62/63; multinational, 2005–2007	VAN iv 1 g q12h	ITT: 372 vs 374; CE: 141 vs 172; ME-S. aureus: 98 vs 109; ME-MRSA: 70 vs 84	Prespecified pooled analysis design; QTcF>500 ms = exclusion; AZT: 60%; PIP-TAZ: 21%; MET: 23%
ATTAIN 2 [26]	MC DB Phase III RCT (5)		VAN iv 1 g q12h	ITT: 377 vs 380; CE: 171 vs 170; ME-S. aureus: 121 vs 105; ME-MRSA: 69 vs 70	

Abbreviations: ref = reference; MC = multicenter; DB = double-blinded; vs = versus; RCT = randomized controlled trial; TLV = telavancin; VAN = vancomycin; PCN = penicillin; AZT = aztreonam; MET = metronidazole; PIP-TAZ = piperacillin-tazobactam; yr = years; q12h = every 12 hours; pts = patients; CrCL = creatinine clearance; QTcF = Fridericia-corrected QT; iv = intravenous; cSSTI = complicated skinn and soft tissue infection; HAP = hospital acquired-pneumonia; ITT = intention-to-treat; CE = clinically evaluable; ME = microbiologically evaluable.

a All studies used telavancin iv at 10 mg/kg q24h, except for FAST 1 (7.5 mg/kg q24h).

b Nafcillin 2 g or oxacillin 2 g or cloxacillin 0.5–1 g.

Regarding cSSTIs, 1112 patients received telavancin, whereas 1117 patients received standard therapy (vancomycin [1090/1117] or antistaphylococcal β-lactam [27/1117]) for 4–14 days. Among 1678 patients with a baseline pathogen, *S. aureus* was isolated in 1353 (81%) subjects, of which 818 (60%) had MRSA infection. Mixed infection (≥2 isolated bacteria) was present in 16% of the ITT populations [23]–[25]. Regarding HAP, 749 and 754 patients received telavancin and vancomycin for 7–21 days, respectively. Among 1089 patients with a baseline pathogen, *S. aureus* was isolated in about 66% of subjects, of which two-thirds had MRSA infection. Infection due to both Gram-positive and Gram-negative bacteria was documented in 27% of patients [26].

### Efficacy in complicated skin and soft tissue infections

**Figure 2:**
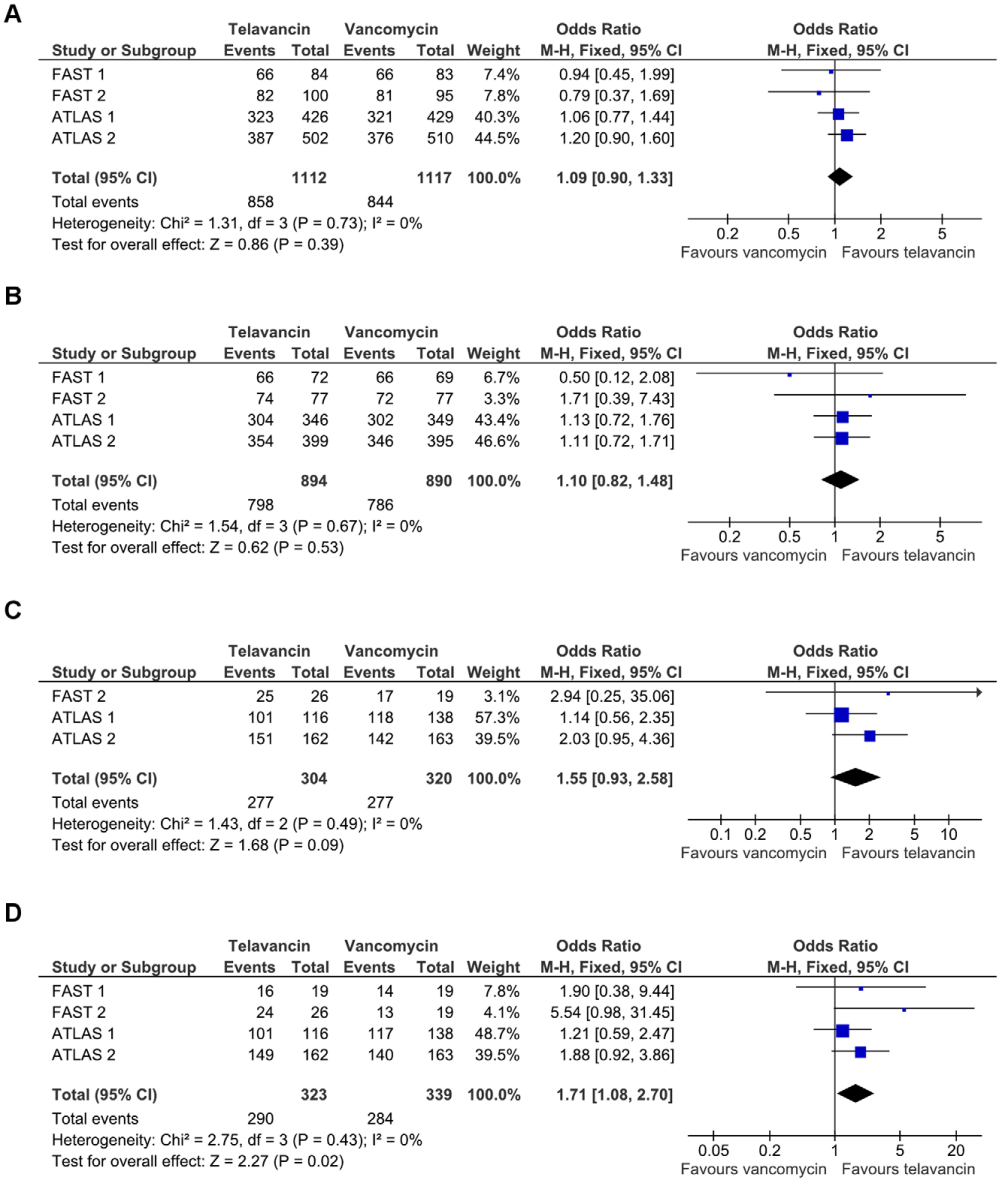
Odds ratios of clinical and microbiologic outcomes with telavancin versus vancomycin in cSSTIs. Panel A: Clinical response in the intention-to-treat population. Panel B: Clinical response in the clinically evaluable population. Panel C: Clinical response in the microbiologically evaluable population with MRSA infection. Panel D: MRSA eradication.

All 4 trials on cSSTIs reported data on clinical response regarding both the ITT and CE populations (Table 2). There was no significant difference regarding clinical success between telavancin and vancomycin in the ITT (2229 patients, 77% vs 76%; OR 1.09 [95% CI 0.90–1.33], FEM; 4 RCTs; figure 2A) or the CE population (1784 patients, 89% vs 88%; OR 1.10 [0.82–1.48], FEM; 4 RCTs; figure 2B) [23]–[25], [27], [28]. When only studies using telavancin at the recommended dose (10 mg/kg) were pooled, similar results were found for the above-mentioned endpoints (data not shown). Clinical response in ME patients with *S. aureus* infection was comparable between telavancin and vancomycin (1027 patients, 90% vs 87%; OR 1.38 [0.93–2.04], FEM; 3 RCTs). However, among subjects with MRSA infection, telavancin was associated with a trend towards higher cure rates (624 patients, 91% vs 87%; OR 1.55 [0.93–2.58], FEM; 3 RCTs; figure 2C) [24], [25], [27], [28].

**Table 2:** Efficacy and safety.

Study ID	Clinical response, n/N (%)	Microbiologic eradication, n/N (%)	Adverse events, n/N (%)
FAST 1	ITT: 66/84 (79) vs 66/83 (80); ITT-S. aureus: 40/50 (80) vs 40/52 (77); ITT-MRSA: 18/22 (82) vs 18/26 (69); CE: 66/72 (92) vs 66/69 (96); ME: 52/56 (93) vs 53/56 (95)	Total: 44/56 (79) vs 46/56 (82); MRSA: 16/19 (84) vs 14/19 (74)	TEAE: 47/84 (56) vs 50/83 (60); SAE: 4/84 (5) vs 9/83 (11); withdrawals: 5/84 (6) vs 4/83 (5); Cr elevation^a^: 7/84 (8) vs 2/83 (2); mortality: NR
FAST 2	ITT: 82/100 (82) vs 81/95 (85); CE: 74/77 (96) vs 72/77 (94); ME: 62/64 (97) vs 53/57 (93); ME-S. aureus: 48/50 (96) vs 37/41 (90); ME-MRSA: 25/26 (96) vs 17/19 (89)	Total: 60/64 (94) vs 47/57 (82); S. aureus: 46/50 (92) vs 32/41 (78); MRSA: 24/26 (92) vs 13/19 (68)	TEAE: 56/100 (56) vs 54/95 (57); SAE: 7/100 (7) vs 3/95 (3); withdrawals: 6/100 (6) vs 3/95 (3); Cr elevation^b^: 3/100 (3) vs 0/95 (0); mortality: 0/100 (0) vs 1/95 (1)
ATLAS 1	ITT: 323/426 (76) vs 321/429 (75); CE: 304/346 (88) vs 302/349 (87); ME: NR; ME-S. aureus^c^: 412/459 (90) vs 414/477 (87); ME-MRSA: 101/116 (87) vs 118/138 (86)	Total: 212/237 (89) vs 219/255 (86); S. aureus^c^: 411/459 (90) vs 414/477 (87); MRSA: 101/116 (87) vs 117/138 (85)	TEAE: 358/426 (84) vs 335/429 (78); SAE: 31/426 (7) vs 27/429 (6); withdrawals^c^: 73/929 (8) vs 53/938 (6); Cr elevation^b^^,^ c: 52/822 (6) vs 19/856 (2); mortality: 5/426 (1) vs 5/429 (1)
ATLAS 2	ITT: 387/502 (77) vs 376/510 (74); CE: 354/399 (89) vs 346/395 (88); ME-MRSA: 151/162 (93) vs 142/163 (87)	Total: 261/290 (90) vs 249/281 (89); MRSA: 149/162 (92) vs 140/163 (86)	TEAE: 377/503 (75) vs 341/509 (67); SAE: 38/503 (8) vs 15/509 (3); mortality: 3/503 (0.6) vs 3/509 (0.6)
ATTAIN 1	ITT: 214/372 (58) vs 221/374 (59); CE: 118/141 (84) vs 138/172 (80); ME^d^: 192/243 (79) vs 182/237 (77); ME-S. aureus: 80/98 (82) vs 81/109 (74); ME-MRSA: 57/70 (81) vs 63/84 (75)	Total: 86/108 (79.6) vs 85/113 (75.2)	TEAE^d^: 616/751 (82) vs 613/752 (82); SAE^d^: 234/751 (31) vs 197/752 (26); withdrawals^d^: 60/751 (8) vs 40/752 (5); Cr elevation^b^^,^ d: 111/716 (16) vs 69/723 (10); mortality: 80/372 (22) vs 62/374 (17)
ATTAIN 2	ITT: 227/377 (60) vs 228/380 (60); CE: 139/171 (81) vs 138/170 (81); ME-S. aureus: 91/121 (75) vs 80/105 (76); ME-MRSA: 47/69 (68) vs 52/70 (74)	Total: 103/135 (76.3) vs 96/124 (77.4)	Mortality: 70/379 (18) vs 78/378 (21)

Abbreviations: ITT = intention-to-treat; CE = clinically evaluable; ME = microbiologically evaluable; TEAE = treatment-emergent adverse events; SAE = serious adverse events; Cr = creatinine.

a Compared to baseline.

b Serum creatinine level ≥1.5 mg/dl at the end-of-therapy visit with an increase of at least 50% or 0.5 mg/dl over the pretreatment level.

c Pooled data from both ATLAS studies.

d Pooled data from both ATTAIN studies.

Overall microbiologic eradication rates were similar between telavancin and standard therapy (1296 patients, 89% vs 86%; OR 1.29 [0.92–1.80], FEM; 4 RCTs), whereas those for MRSA were significantly higher with telavancin (662 strains, 90% vs 84%; OR 1.71 [1.08–2.70], FEM; 4 RCTs; figure 2D) [23]–[25], [27], [28]. Median duration of treatment was identical between treatment groups in FAST 1 study (7 days) [23], whereas in ATLAS studies it was 1 day shorter in the telavancin group (10 vs 11, and 8 vs 9 days) [25].

### Efficacy in hospital-acquired pneumonia

Two methodologically identical RCTs compared telavancin to vancomycin in the setting of HAP, using a non-inferiority margin of 20%. Telavancin proved to be non-inferior to vancomycin on the basis of clinical response in each trial. The prespecified pooled analysis of these trials failed to show superiority of telavancin in patients with MRSA infection (293 patients, 74.8% vs 74.7%). Mortality rates with telavancin versus vancomycin were 22% versus 17% (95% CI of the difference [−0.7–10.6]) in the first trial, and 18% versus 21% (95% CI of the difference [−7.8–3.5]) in the second trial. Subgroup analyses showed higher cure rates with telavancin in patients with monomicrobial *S. aureus* infection (298 patients, 84.2% vs 74.3%) or pneumonia caused by *S. aureus* with vancomycin MIC ≥1 µg/ml (190 patients, 87% vs 74%). The median duration of treatment was comparable between treatment groups in each trial (9–10 days) [26].

### Safety and tolerability

**Figure 3:**
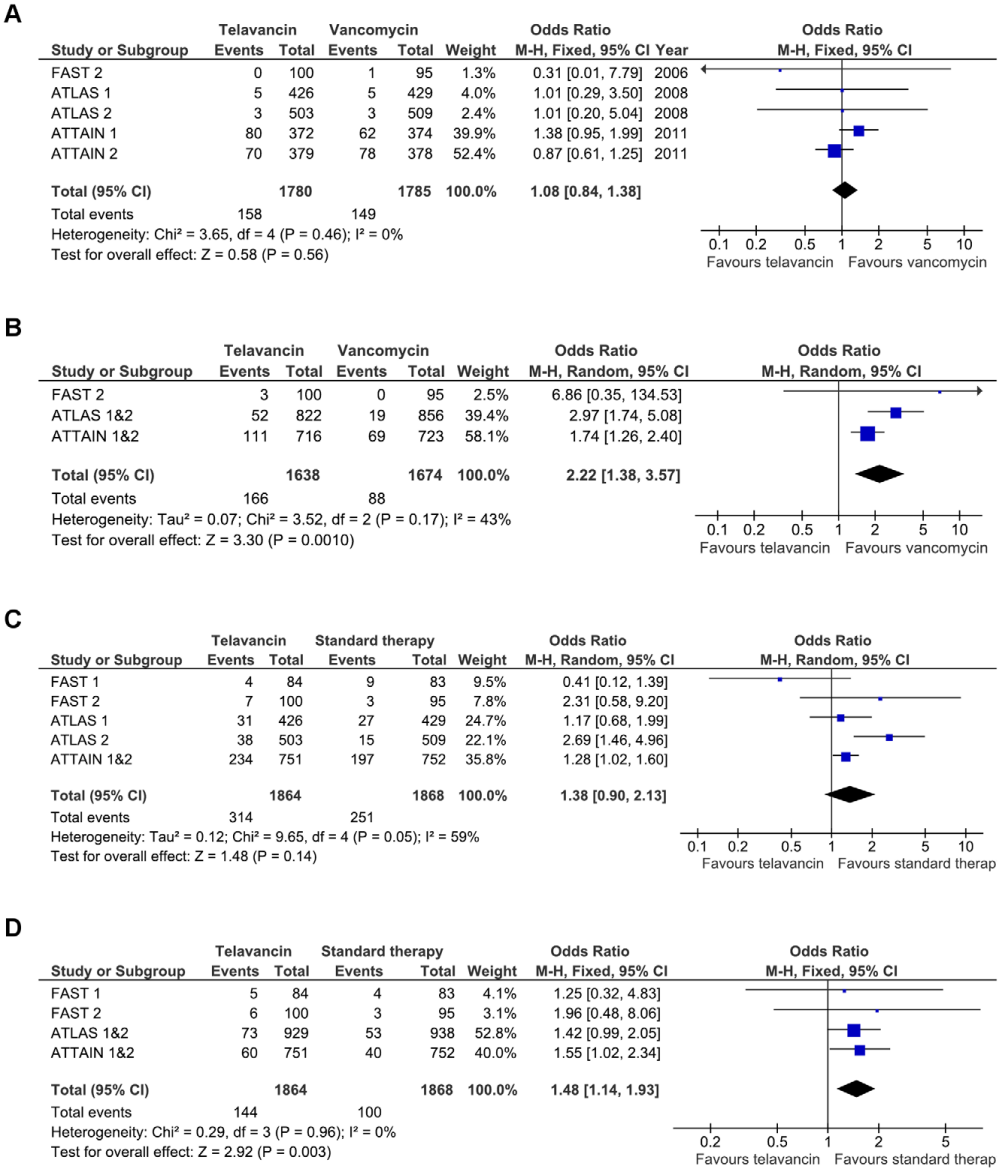
Odds ratios of adverse events with telavancin versus vancomycin in cSSTIs and HAP. Panel A: All-cause mortality. Panel B: Elevation in serum creatinine concentration. Panel C: Serious adverse events. Panel D: Adverse-event related withdrawals.

The safety analysis was based on synthesis of trial data from both cSSTIs and HAP (Table 3). Overall, mortality rates were comparable between telavancin and vancomycin (3565 patients, 8.9% vs 8.3%; OR 1.08 [0.84–1.38], FEM; 5 RCTs; figure 3A) [23]–[28]. Clinically significant increases in serum creatinine were more frequently observed with telavancin compared to vancomycin (3312 patients, 10% vs 5%; OR 2.22 [1.38–3.57], REM; 5 RCTs; figure 3B) [24]–[28]. Overall AE (3732 patients, 78% vs 75%; OR 1.20 [0.97–1.49], REM; 6 RCTs) showed a trend towards higher rates with telavancin, whereas serious AE (3732 patients, 17% vs 13%; OR 1.38 [0.90–2.13], REM; 6 RCTs; figure 3C) were comparable between telavancin and vancomycin recipients. AE-related withdrawals were more common in the telavancin group (3732 patients, 8% vs 5%; OR 1.48 [1.14–1.93], FEM; 6 RCTs; figure 3D) [23]–[28]. When only studies using telavancin 10 mg/kg were analyzed, telavancin recipients also showed higher rates of overall AE (3565 patients, 79% vs 75%; OR 1.25 [1.01–1.55], REM; 5 RCTs) and serious AE (3565 patients, 17% vs 14%; OR 1.53 [1.05–2.24], REM; 5 RCTs) [24]–[28].

**Table 3:** Adverse events and laboratory abnormalities for pooled cSSTIs and HAP studies.^a^

AE, n/N (%)	Telavancin	Vancomycin	OR (95% CI)
Overall AE	1454/1864 (78)	1393/1868 (74.6)	1.20 (0.97–1.49)
Serious AE	314/1864 (16.8)	251/1868 (13.4)	1.38 (0.90–2.13)
Withdrawals	144/1864 (7.7)	100/1868 (5.4)	1.48 (1.14–1.93)
Nausea	318/1864 (17.1)	190/1868 (10.2)	1.88 (1.54–2.29)
Vomiting	143/1113 (12.8)	78/1116 (7)	1.97 (1.47–2.63)
Taste disturbance	325/1029 (31.6)	62/1033 (6)	7.37 (5.52–9.85)
Diarrhoea	73/1029 (7.1)	81/1033 (7.8)	0.90 (0.65–1.25)
Constipation	174/1864 (9.3)	144/1868 (7.7)	1.12 (0.72–1.74)
Insomnia	137/1780 (7.7)	136/1785 (7.6)	1.14 (0.62–2.11)
Pruritus	34/1029 (3.3)	68/1033 (6.6)	0.48 (0.32–0.74)
Headache	147/1113 (13.2)	132/1116 (11.8)	1.14 (0.89–1.47)
Chills	47/1029 (4.6)	23/1033 (2.2)	2.10 (1.27–3.48)
Cr elevation	166/1638 (10.1)	88/1674 (5.3)	2.22 (1.38–3.57)
Hypokalemia	73/1528 (4.8)	44/1521 (2.9)	1.91 (0.91–4.00)
AST increase	36/1045 (3.4)	39/1084 (3.6)	0.93 (0.43–2.04)
ALT increase	38/1101 (3.5)	61/1165 (5.2)	0.64 (0.42–0.97)
QTcF increase^b^	59/1560 (3.8)	49/1578 (3.1)	1.24 (0.84–1.83)
Anemia	66/1052 (6.3)	65/1058 (6.1)	1.01 (0.71–1.46)
Leukopenia	12/1006 (1.2)	19/989 (1.9)	0.62 (0.30–1.28)
Platelet decrease^c^	8/1064 (0.8)	10/1110 (0.9)	0.87 (0.35–2.17)

a The FAST 1 study is included in the analysis.

b >60 ms.

c <75×109/L.

## Discussion

Telavancin was non-inferior to vancomycin in the treatment of patients with cSSTIs and HAP in large Phase III RCTs [25]–[28]. Our meta-analysis indicated that telavancin was associated with significantly higher eradication rates and a trend towards better clinical response among patients with MRSA skin and soft tissue infections. All-cause mortality was similar between telavancin- and vancomycin-treated patients with cSSTIs or HAP. On the other hand, telavancin at the recommended dose was associated with higher rates of treatment-emergent adverse events, serious adverse events, adverse event-related withdrawals, and elevations in serum creatinine.

Regarding cSSTIs, our analysis found a trend towards higher cure rates with telavancin compared to standard therapy in patients with MRSA infections; microbiologic eradication was also higher with telavancin among these patients. It should be noted that the RCTs excluded patients with chronic diabetic foot, necrotizing fasciitis or osteomyelitis [23]–[25]. The more potent and rapidly bactericidal activity of telavancin against *S. aureus* compared to vancomycin may be an explanation for the better performance of this lipoglycopeptide antibiotic [29], [30]. The MIC_90_ values of telavancin against MRSA (0.25–0.5 µg/ml) in the cSSTIs trials were two- to four-fold lower than those of vancomycin (1 µg/ml) [23]–[25], [31]. A multiple-comparison meta-analysis also found higher success rates with telavancin, dalbavancin or linezolid compared to vancomycin in MRSA cSSTIs [32].

A number of post-hoc analyses of the ATLAS studies on cSSTIs have been performed [33]–[35]. Stryjewski et al. reported similar clinical response rates in patients with different types of cSSTIs (major abscess, infective cellulitis, wound infection), including infections caused by MRSA [33]. Clinical efficacy was also similar with telavancin or vancomycin in both obese and non-obese patients [34]. In addition, a recent post-hoc analysis addressing the new FDA guidance for cSSTIs found that telavancin and vancomycin demonstrated comparable efficacy in patients with baseline lesion ≥75 cm^2^ [35], [36]. Despite the limitations of post-hoc analyses, the consistency of their findings indicates that telavancin is effective in the treatment of cSSTIs and could be an alternative to vancomycin for MRSA infections.

Regarding HAP, clinical cure rates were similar between telavancin and vancomycin. The 20% non-inferiority margin of the ATTAIN studies is controversial; nevertheless, the lower bound of the 95% CI around the difference between treatments in clinical response exceeded −10% for both ITT and CE patients. The pooled mortality was comparable between treatment groups. However, an individual trial indicated a trend towards higher mortality among telavancin-recipients. It is not clear whether this finding is an issue of efficacy or safety of telavancin and warrants further investigation [16], [26]. Regarding VAP, post-hoc analyses showed that clinical response with telavancin and vancomycin was not significantly different in the ITT (427 patients, 49% vs 53%), CE (135 patients, 80% vs 66%) and ME (118 patients, 78% vs 61%) populations [37]. Of note, recent studies suggest that linezolid is superior to vancomycin in terms of clinical response in nosocomial pneumonia [38], [39]. In this regard, the use of telavancin in HAP may be considered in cases of infection due to Gram-positive organisms that are resistant to common antimicrobial agents.

An issue regarding ATTAIN studies that should be mentioned is the fact that 34% of patients with available relevant data had trough vancomycin levels <10 µg/ml [26]. Although firm prospective data are not available, current guidelines suggest that trough levels of vancomycin be 15–20 µg/ml [40], [41]. In this regard, the trials' investigators reported that, in multiple subgroup analyses stratifying patients by vancomycin serum trough level, they found lower clinical response rates and higher mortality and nephrotoxicity rates in the highest trough group [42]. In addition, it has been suggested that vancomycin MIC values in the higher levels of the susceptible range (1–2 µg/ml) are associated with adverse clinical outcomes [43]; in post-hoc analysis of the ATTAIN studies, telavancin was associated with higher clinical success rates than vancomycin among patients infected by isolates with vancomycin MIC ≥1 µg/ml [16], [26]. The role of telavancin in the treatment of patients with infections by *S. aureus* isolates with “higher” vancomycin MIC warrants further investigation.

The analyzed data raise concerns regarding the safety profile of telavancin. The most commonly reported AE in the telavancin group were foamy urine, nausea, vomiting, taste disturbance and headache; most of them were mild to moderate in intensity and reversible [23]–[26]. In our analysis, telavancin was associated with higher rates of serious AE (17%) and AE-related withdrawals (8%). Most common serious AE in the telavancin group were renal AE (including acute renal failure, increased serum creatinine and renal insufficiency), anaemia, sepsis and multi-organ failure. The most common AE that led to discontinuation of telavancin therapy were renal AE, nausea and vomiting [37], [44].

In addition, elevation in serum creatinine concentration was more frequently observed with telavancin; the renal impairment was generally reversible [23]–[26]. A recent retrospective cohort study found that 7 of 21 patients (33%) developed acute renal insufficiency after a median time of 9 days on telavancin. Most of these patients had comorbidities and received telavancin for non-approved indications [45]. Therefore, monitoring of serum creatinine concentration seems warranted, and the administration of telavancin in patients with risk factors for renal AE should be based on a benefit-risk approach. Telavancin also has an effect on cardiac repolarization. An early study on healthy volunteers found a placebo-corrected mean change in QTcF 4.5 ms with telavancin 15 mg/kg/24 h for three days [46]. In the cSSTIs and HAP trials, respectively, the mean change from baseline in QTcF was 7 ms and 4 ms longer in the telavancin group [44]. However, no cardiac AEs associated with QT prolongation were reported [23]–[26].

Our study has specific limitations that need to be considered in the interpretation of our findings. First, only a few studies have been conducted on this issue and the Phase III trials are those that mostly influenced our results; however, the pooled sample size is relatively large (2229 patients with cSSTIs and 1503 patients with HAP) [23]–[26]. Furthermore, one of the analyzed studies was a Phase II trial evaluating telavancin at a lower than the recommended dose; however, there was no difference in our results when it was excluded from analysis [23].

In conclusion, telavancin is a potent antimicrobial agent against Gram-positive infections that proved to be non-inferior to vancomycin in the treatment of patients with cSSTIs or HAP, and might be a therapeutic alternative in cases of difficult-to-treat MRSA infections. However, the potential for nephrotoxicity of this antibiotic should be taken under consideration. Additional randomized controlled trials, optimized to assess mortality, are warranted to better clarify the role of telavancin in the treatment of patients with hospital-acquired pneumonia.
